# The Tumor Microenvironment Drives Intrahepatic Cholangiocarcinoma Progression

**DOI:** 10.3390/ijms23084187

**Published:** 2022-04-10

**Authors:** Serena Mancarella, Grazia Serino, Sergio Coletta, Raffaele Armentano, Francesco Dituri, Francesco Ardito, Andrea Ruzzenente, Isabel Fabregat, Gianluigi Giannelli

**Affiliations:** 1 National Institute of Gastroenterology “S. De Bellis”, IRCCS Research Hospital, 70013 Castellana Grotte, Italy; grazia.serino@irccsdebellis.it (G.S.); sergio.coletta@irccsdebellis.it (S.C.); raffaele.armentano@irccsdebellis.it (R.A.); francesco.dituri@irccsdebellis.it (F.D.); 2 Hepatobiliary Surgery Unit, Foundation “Policlinico Universitario A. Gemelli”, IRCCS, Catholic University, 00168 Rome, Italy; francesco.ardito@policlinicogemelli.it; 3 Department of Surgery, General and Hepatobiliary Surgery, University Hospital G.B. Rossi, University and Hospital Trust of Verona, 37126 Verona, Italy; andrea.ruzzenente@univr.it; 4 Oncobell Program, Bellvitge Biomedical Research Institute (IDIBELL), CIBEREHD and University of Barcelona, 08908 L’Hospitalet de Llobregat, Spain; ifabregat@idibell.cat

**Keywords:** intrahepatic cholangiocarcinoma, NOTCH1, HuCCT1-xenograft mouse model, gene expression, microenvironment

## Abstract

Intrahepatic cholangiocarcinoma (iCCA) is a highly aggressive cancer with limited therapeutic options and short overall survival. iCCA is characterized by a strong desmoplastic reaction in the surrounding ecosystem that likely affects tumoral progression. Overexpression of the Notch pathway is implicated in iCCA development and progression. Our aim was to investigate the effectiveness of Crenigacestat, a selective inhibitor of NOTCH1 signaling, against the cross-talk between cancer cells and the surrounding ecosystem in an in vivo HuCCT1-xenograft model. In the present study, a transcriptomic analysis approach, validated by Western blotting and qRT-PCR on iCCA tumor masses treated with Crenigacestat, was used to study the molecular pathways responsive to drug treatment. Our results indicate that Crenigacestat significantly inhibited NOTCH1 and HES1, whereas tumor progression was not affected. In addition, the drug triggered a strong immune response and blocked neovascularization in the tumor ecosystem of the HuCCT1-xenograft model without affecting the occurrence of fibrotic reactions. Therefore, although these data need further investigation, our observations confirm that Crenigacestat selectively targets NOTCH1 and that the desmoplastic response in iCCA likely plays a key role in both drug effectiveness and tumor progression.

## 1. Introduction

Intrahepatic cholangiocarcinoma (iCCA) has now become the second most common primary liver malignancy after hepatocellular carcinoma (HCC) [1], generating a renewed interest in this disease among the scientific community. The global incidence and mortality rates of iCCA are the highest in East Asia, but there is also an increase in western countries, including Italy [2,3]. The prognosis is extremely dismal because the majority of iCCA patients present with advanced-stage disease, and 75% of patients die within 1 year of diagnosis, with the 5-year survival rate being below 5% [4]. Surgical resection following neoadjuvant chemoradiation is the only treatment with curative intent for achieving long-term survival outcomes, but it is limited to only 20–30% of cases with early-stage disease [1]. For patients with advanced-stage or unresectable tumors, combined administration of chemotherapeutic agents such as Gemcitabine and Cisplatin is the first-line systemic therapy. However, this current standard-of-care chemotherapy regimen shows only partial benefit as it induces little improvement in progression-free survival [5]. Current treatment options have limited efficiency since adaptation mechanisms mediated by the tumor microenvironment (TME) contribute to drug resistance. In particular, solid tumor cancer cells typically interact with surrounding components, promoting cell proliferation and survival [6] and regulating the response to chemotherapy. Numerous studies have shown that iCCA is characterized by a prominent desmoplastic stroma with a dense extracellular matrix (ECM), mainly enriched by cancer-associated fibroblasts (CAFs). The abundant tumor immune microenvironment is populated by T lymphocytes, B lymphocytes, NK cells, natural killer T cells and tumor-associated macrophages or TAM, endothelial cells (EC) and their secretomes, exosomes, and other soluble factors responsible for driving tumor angiogenesis, growth, and metastasis [7,8,9,10]. CAFs, identified by a strong α-smooth muscle actin (α-SMA) expression, communicate extensively with the CCA cells and immune cells, enhancing the malignant phenotype [8]. In addition, CAFs produce angiogenic factors to induce tube formation and recruit infiltrating immune cells, promoting tumor cell growth, angiogenesis, matrix turnover, and suppression of the adaptive immune response [6]. Overall, the mutual interaction between tumor cells and their microenvironment involves the activation of multiple pathways promoting iCCA pathogenesis [11]. Among these, the NOTCH canonical signaling pathway is known to be a key player in direct cell–cell communication among tumor cells and each of the components of the microenvironment. In the last years, a significant body of literature has supported the role of NOTCH in cancer [12,13,14,15]. Overall, NOTCH signaling is activated directly by the engagement of one of the four transmembrane isotypes of the NOTCH receptor (NOTCH 1–4) with one of the five ligands, Jagged-1, -2 and Delta-like-1, -3, -4, which are also transmembrane proteins of the cell surface. Following the interaction, gamma-secretase, a multi-subunit protease complex, intervenes, releasing the active fragment “NOTCH Intra Cellular Domain” (NICD) from the cytoplasmic membrane. NICD moves to the nucleus and, by binding to a transcriptional complex, induces the transcription of target genes, such as those belonging to the Hairy/enhancer of the split (HES) family [13,14]. NOTCH has been identified as having the potential to induce CCA when transgenically overexpressed [16]. In addition, some investigations reported aberrant NOTCH1 in iCCA tissue, contributing to tumor growth [17,18,19]. Recently, we demonstrated that Crenigacestat, a new gamma secretase inhibitor (GSI), was able to reduce the growth of tumors expressing high levels of CD90 [20] and reduced NOTCH-dependent angiogenesis in a patient-derived xenograft (PDX) mice model of iCCA [19]. Particularly, Crenigacestat was able to selectively inhibit NOTCH1 in vitro and in the PDX model, reducing tumor progression by blocking new vessel formation in the surrounding desmoplastic reaction tissue. However, the cross-talk between iCCA cells and the surrounding ecosystem is likely responsible for the sensitivity to treatment as well as the tumor development.

The aim of the study was to investigate in further detail the effectiveness of Crenigacestat on the stroma/tumor interaction in iCCA tumoral progression following HuCCT1-xenograft implantation.

## 2. Results

### 2.1. Crenigacestat Treatment in the HuCCT1-Xenograft Mouse Model

**Figure 1:**
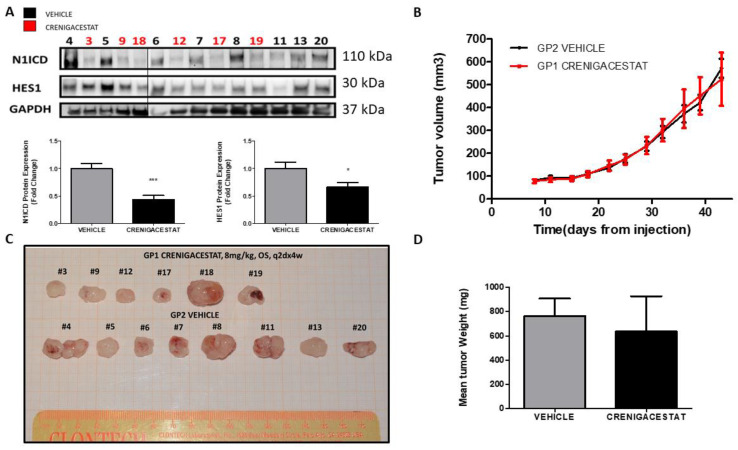
Crenigacestat does not reduce iCCA tumor progression but inhibits Notch1 pathway in the iCCA HuCCT1-xenograft model. (**A**) Western blotting shows significant inhibition of N1ICD (*** *p* < 0.001) and HES1 (* *p* < 0.05) expression in treated HuCCT1-xenograft tissues related to GAPDH expression for each respective tissue. The semi-quantitative evaluation by densitometry analysis of protein bands is shown in the graphs, comparing the average value of the levels of N1ICD and HES1 among all the mice treated with Crenigacestat normalized with respect to vehicle. (**B**) Crenigacestat, compared with vehicle, has no effect on tumor progression (**C**,**D**), nor does it reduce the weight of the iCCA masses in the HuCCT1-xenograft model. Explanted tumor masses of HuCCT1-xenograft mice *n* = 8 for vehicle treatment, *n* = 6 for Crenigacestat treatment.

In our recent study [19], we provided evidence that Crenigacestat treatment reduced iCCA tumor progression, targeting NOTCH in a PDX model. In this current study, Crenigacestat, used at 8 mg/kg, significantly inhibited the NOTCH1 pathway, as demonstrated by Western blotting analysis on tissue extracts. Densitometric analysis showed a significant inhibition of the NOTCH1 intracellular domain (N1ICD) (*p* < 0.001) and HES1 (*p* < 0.05) in Crenigacestat-treated HuCCT1-xenograft mice (Figure 1A). Surprisingly, the drug did not exert any effect on tumor growth nor on tumor weight as compared to the vehicle (Figure 1B–D); nevertheless, it inhibited the NOTCH1 pathway. These results indicate that GSI interferes with NOTCH activation, as expected, and that in a HuCCT1-xenograft model, Crenigacestat inhibits NOTCH1 signaling but does not display any effect on tumor growth.

### 2.2. Gene Expression Analysis of the HuCCT1-Xenograft Mouse Model

**Figure 2:**
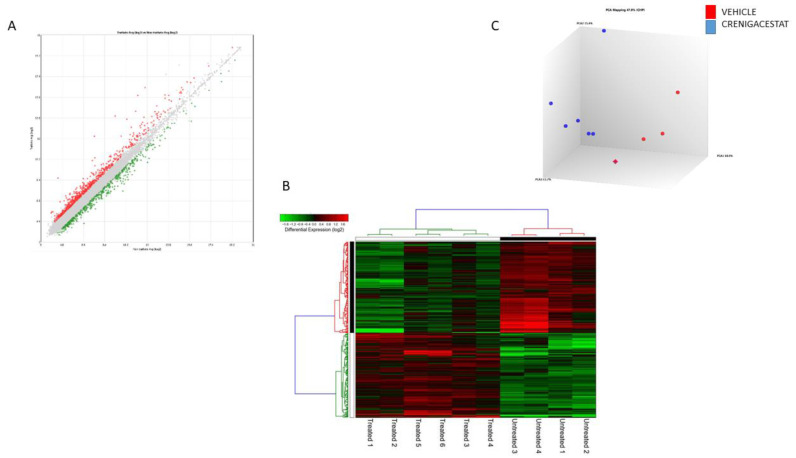
Global gene expression profile in HuCCT1-xenograft model treated and untreated with Crenigacestat reveals molecular changes. (**A**) PCA clearly shows the separation between treated and untreated mice. (**B**) The separation between treated and untreated mice is further confirmed by the analysis of unsupervised hierarchical clustering. Each row represents a gene, and each column represents a sample. A color code represents the relative intensity of the expression signal, where red and green indicate high and low expression, respectively, according to the scale shown on the top. (**C**) Scatterplot of all assayed probes shows the distribution of differentially expressed genes based on the expression data of treated and untreated mice, applying a cut-off *p*-value threshold lower than 0.005 and a fold change of 1.5. The X-axis represents the averaged log2 signal of untreated samples, and the Y-axis represents the averaged log2 signal of treated samples.

In order to evaluate the gene profile modulated by Crenigacestat treatment in the HuCCT1-xenograft mouse model, we performed transcriptomic analysis, comparing the expression profiles of tissues from HuCCT1-xenograft mice treated with Crenigacestat or with the vehicle. We identified 596 differentially expressed genes, 286 upregulated and 310 downregulated after treatment with Crenigacestat (Fold-change ± 1.5, *p*-value 0.05) (Figure 2A, Supplementary Table S1). Unsupervised hierarchical clustering analysis and principal component analysis showed distinct gene expression patterns between Crenigacestat-treated and untreated mice (Figure 2B,C).

### 2.3. Crenigacestat Affects the Notch Pathway in the HuCCT1-Xenograft Mice Model

Then, we investigated interactions between Crenigacestat-modulated genes in the HuCCT1-xenograft mice model by ingenuity pathway analysis (IPA). A total of 24 significant molecular networks were identified; one of the top-ranked networks selected by this analysis consists of 24 DEGs, whose top function includes cancer, cell morphology, and tissue development (Table 1).

**Figure 3:**
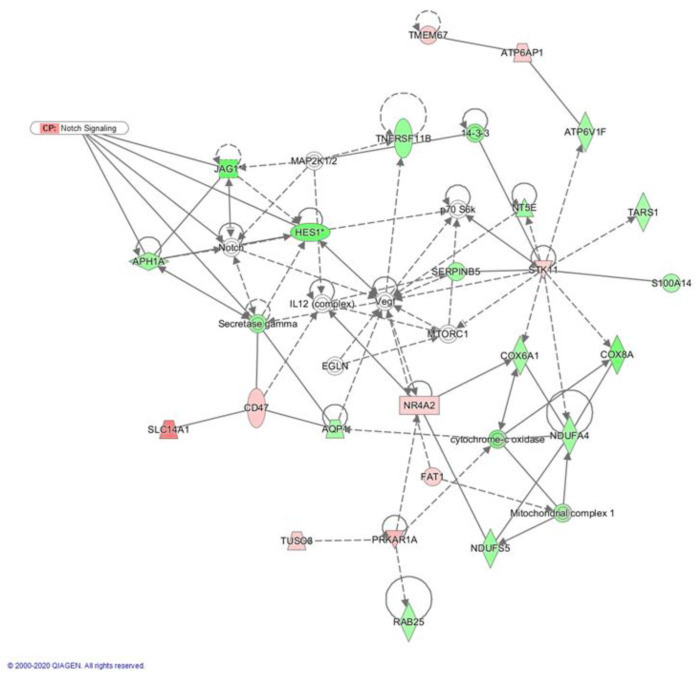
Bioinformatic analysis of networks with ingenuity pathway analysis (IPA). The analysis reports annotated interactions between genes modulated by Crenigacestat treatment in the HuCCT1-xenograft mouse model. The top-ranked network showing a high degree of interconnectivity between genes and the IPA functional category of this network was cancer, cell morphology, and tissue development. The Figure shows that Crenigacestat downregulated key Notch signaling genes, confirming the specificity of the treatment. Up- (red) and down- (green) regulated genes are indicated.

Interestingly, pathway analysis of this network revealed a clear involvement of the Notch pathway in the downregulation of *JAG1, HES1, APH1A*, and *Gamma secretase* (Figure 3), indicating that Crenigacestat in vivo significantly reduces the Notch pathway.

### 2.4. Biological Functions Regulated by Crenigacestat Treatment in the HuCCT1-Xenograft Mice Model

**Figure 4:**
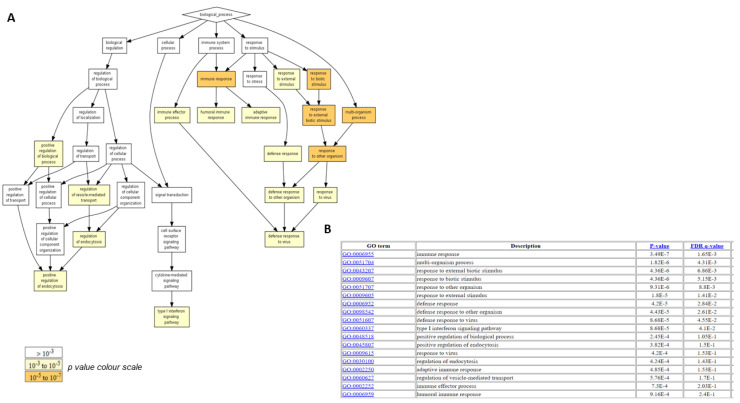
Gene ontology functional enrichment analysis of differentially expressed genes modulated by Crenigacestat. The deregulated genes after Crenigacestat treatment in HuCCT1-xenograft mouse model showed few enriched biological processes linked mainly to the immune response, response to external stimuli, and regulation of vesicle-mediated transport. Enriched GO terms are visualized using (**A**) a directed acyclic graph (DAG) graphic representation with color coding reflecting their degree of enrichment and (**B**) a table with the corresponding *p*-value and FDR q-value for each term.

To identify the Crenigacestat treatment-enriched biological processes in the HuCCT1-xenograft mice model, we carried out gene ontology (GO) enrichment analysis on DEGs. Results showed that few biological processes were significantly enriched; the most significant of these were mainly associated with the immune response, response to external stimuli and regulation of vesicle-mediated transport (Figure 4).

**Figure 5:**
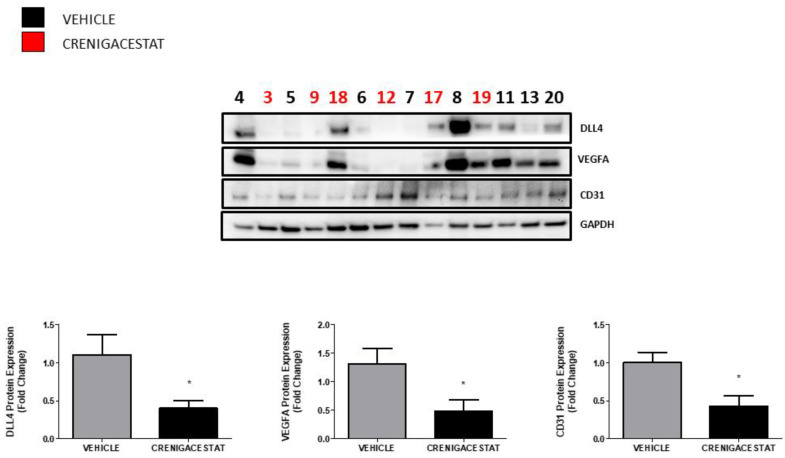
Crenigacestat inhibits angiogenesis in HuCCT1-xenograft model. Western blotting identified a significant downregulation of CD31 (* *p* < 0.05), DLL4 (* *p* < 0.05), and VEGFA (* *p* < 0.05) protein expression, involved in angiogenesis, in treated HuCCT1-xenograft tissues normalized to GAPDH protein expression for each tissue. The graphs show the semi-quantitative evaluation by densitometry analysis of protein, comparing the average intensity value of the bands of CD31, DLL4, and VEGFA among all the mice treated with Crenigacestat versus vehicle. Tumor masses of HuCCT1-xenograft mice *n* = 8 for vehicle treatment, *n* = 6 for LY3039478 treatment.

In our previous study conducted on the PDX mice model, one of the most significant upstream regulators predicted by IPA was *VEGFA* (vascular endothelial growth factor A), which was inhibited [19], together with DLL4 and CD31. In order to confirm these data in the HuCCT1-xenograft mice model, we analyzed the expression of this same protein. Semi-quantitative evaluation of Western blotting corroborated, as shown in Figure 5, that Crenigacestat blocks the neovascularization, followed by a significant reduction of CD31 (*p* < 0.05), delta-Like canonical Notch ligand 4 (DLL4) (*p* < 0.05), and VEGFA (*p* < 0.05) proteins.

To prove this hypothesis, we looked for other networks generated by the IPA functional analysis. A network generated by the dataset of Crenigacestat-modulated genes in the HuCCT1-xenograft mouse model had the following top functions: connective tissue development and function, connective tissue disorders, and organism injury and abnormalities.

**Figure 6:**
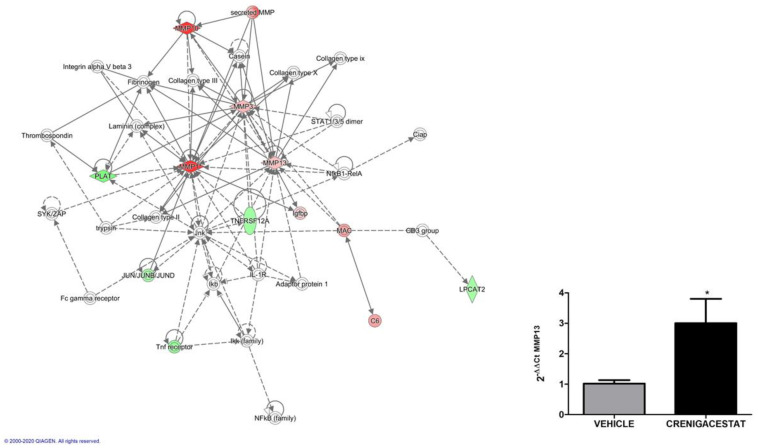
Network analysis with ingenuity pathway analysis (IPA) and microarray validation with real-time PCR. The analysis reports annotated interactions between genes modulated by Crenigacestat treatment in the HuCCT1-xenograft mouse model. The IPA functional category of this network was connective tissue development and function, connective tissue disorders, and organism injury and abnormalities. The Figure shows that even though Crenigacestat affected some metalloprotease (MMPs) genes, key elements of extracellular matrix such as fibrinogen, some collagens, laminins, and integrin were not altered by treatment. Up- (red) and down- (green) regulated genes are indicated. Blank nodes were suggested by IPA as potential targets functionally linked to deregulated genes. Validation of the *MMP13* gene identified a significant (* *p* < 0.05) up-regulation of *MMP13* by quantitative real-time PCR.

As shown in Figure 6, Crenigacestat treatment acts on some genes of the extracellular matrix, including several metalloproteases (*MMP1, MMP3, MMP10, MMP12, and MMP13*). Particularly, MMP13 was already found to be modulated after Crenigacestat treatment in the PDX model [19], but contrariwise, an increased expression was observed by quantitative reverse transcriptase PCR (qRT-PCR) in the HuCCT1-xenograft model.

**Figure 7:**
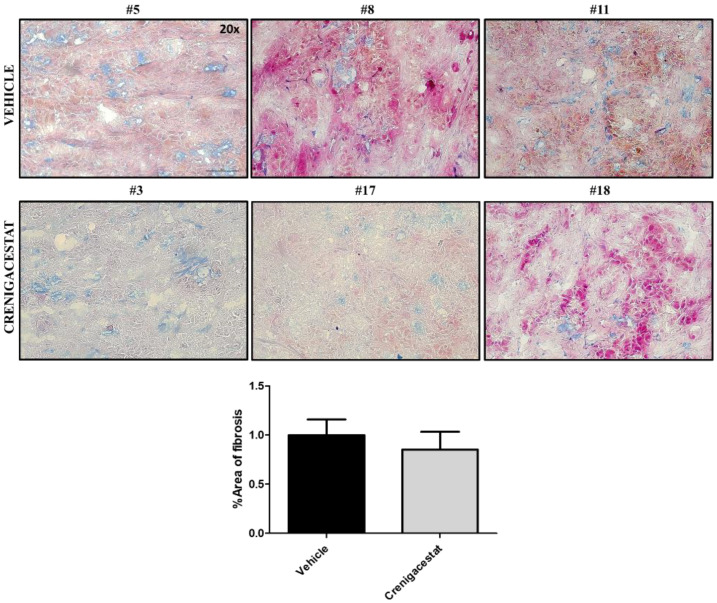
Crenigacestat has no effect on fibrosis in the HuCCT1-xenograft model. Tissue sections were stained using Azan-Mallory’s trichrome staining to highlight collagen fibers. Representative images of treated and untreated animals reveal collagen in aniline blue, ordinary cytoplasm in orange G, and nucleus in acid fuchsin in both conditions. Quantitative analysis of blue stained area to the total area in HuCCT1-xenograft mice treated with vehicle (*n* = 8) and Crenigacestat (*n* = 6) shows no significant differences between the two groups. These results indicate that Crenigacestat is not able to modulate fibrosis in this model. The images were acquired at 20× magnification. Scale bar represents 50 μm.

**Figure 8:**
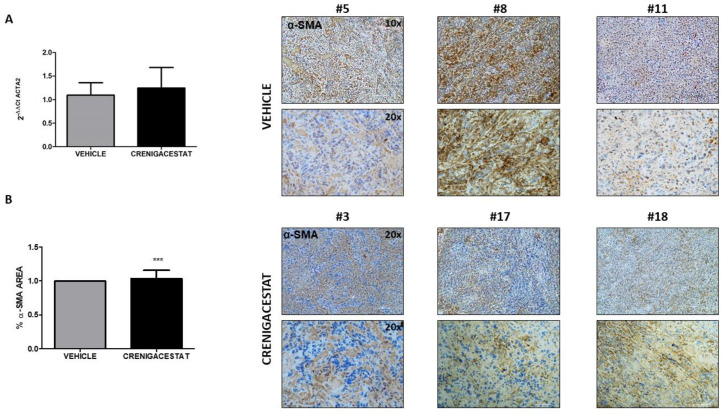
Crenigacestat has no effect on α-SMA expression in the HuCCT1-xenograft model. (**A**) RNA and (**B**) protein expression of murine α-SMA were analyzed in HuCCT1-xenograft tissues in Crenigacestat and vehicle-treated mice. Representative images of treated (*n* = 6) and untreated (*n* = 8) animals reveal no differences in tissues in both groups, confirmed by quantitative analysis of positive staining to the total area in tissues of HuCCT1-xenograft mice. These results indicate that Crenigacestat is not able to modulate fibrosis in this model (*** *p* < 0.001). The images were acquired at 10× and 20× magnification. Scale bar represents 100 μm and 50 μm, respectively.

Surprisingly, the IPA functional analysis revealed that fibrinogen, some collagens, laminins, and integrins had not been altered by treatment. To confirm the relevance of the IPA network analysis, we quantified, in frozen specimens, the severity of the fibrosis status in treated and untreated HuCCT1-xenograft mice by Azan-Mallory staining. Consistently, a panel in Figure 7 shows a moderate presence of fibrous connective tissue, and Figure 8 shows no difference in terms of gene and protein expression of α-SMA, used as a marker of activated stromal cells, in treated compared to untreated mice. These results indicate that Crenigacestat does not influence ECM deposition and stromal components in this animal model.

**Figure 9:**
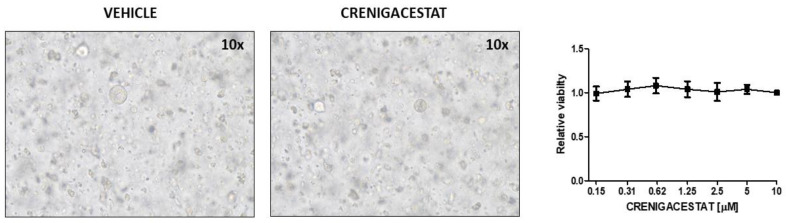
Crenigacestat has no effect on iCCA-PDOs. Phase-contrast images of two wells treated with DMSO or Crenigacestat (10 µM). Dose–response curves of Crenigacestat after 15 days show no difference in relative viability. Viability values are expressed as mean ± SEM.

To further support the hypothesis that the microenvironment is essential for Crenigacestat effectiveness on iCCA progression, we challenged patient-derived organoids (PDOs) resembling near-physiological architectures because of the contextual presence of Matrigel as a three-dimensional ECM structure with different concentrations of Crenigacestat. As reported in Figure 9, drug treatment does not affect the growth of iCCA PDOs (Figure 9).

## 3. Discussion

New, improved therapeutic strategies for iCCA patients are urgently needed. Persistent activation of Notch signaling has been reported to induce the onset of iCCA [14,15,16,17,18,19,21]. Additionally, previous studies have reported high expression levels of Notch1 in human iCCA specimens compared to peritumoral tissue [17,19], highlighting the role of this pathway in cholangiocarcinogenesis. In line with these observations, in this work, we report the effect of Crenigacestat on Notch signaling, without any significant reduction of volume and weight in the treated iCCA, in a HuCCT1-xenograft mouse model. These data are consistent with our recent study reporting an effect of Crenigacestat on tumors originated by CD90 transfected HuCCT1, but not on wild-type cells [20]. Consequently, we performed gene expression profiling in explanted iCCA masses, while IPA analysis showed a robust involvement of the NOTCH pathway and downstream targets such as *JAG1, HES1, APH1A*, and γ-secretase, as expected. Consistently with our previous work [19], Crenigacestat blocked Notch signaling, demonstrating downregulation of the protein expression of NOTCH1 and HES1.

Although this in vivo model offers the opportunity to directly predict drug sensitivity as compared to the in vitro model, it has some limitations, such as the influence of the murine microenvironment and the host immune response. Surprisingly, although these animal models are defective in T-cell-mediated immunity [22], by GO enrichment analysis, we identified some biological processes significantly associated with the immune response, response to external stimuli, and regulation of vesicle-mediated transport, suggesting that Crenigacestat is able to modulate the activity of signaling pathways. Thus, in this model, it triggered an important immune response and a dynamic cross-talk between tumor cells and host surrounding tissue.

Like in our previous findings in PDX [22], validation of the HuCCT1-xenograft mouse model by Western blotting confirmed downregulation of CD31, DLL4, and VEGFA, commonly associated with angiogenesis. These results consistently demonstrate the effects of Crenigacestat on neovascularization.

The tumor stroma is an essential component in the progression of iCCA, as it provides trophic signals involved in tumor progression through intense extracellular matrix remodeling and inflammation [7,23,24]. In support of this hypothesis, IPA functional analysis generated a network showing that Crenigacestat treatment acts by upregulating several metalloproteases such as *MMP1, MMP3, MMP10, MMP12*, and *MMP13*, probably triggered by host inflammatory response [25,26]. However, no modulation of the ECM compounds, such as fibrinogen, some collagens, laminins, and integrins, was noted, suggesting that specific MMPs in this model have a role in regulating the inflammatory processes. Furthermore, these data are consistent with the lack of Crenigacestat effectiveness on stromal cell activation responsible for ECM production and deposition, as documented by α-SMA expression levels in treated and control mice.

ICCA is characterized by a highly desmoplastic tumor microenvironment enriched by cancer-associated fibroblasts (CAFs) [6,27]. Particularly, once fibroblasts are recruited via various types of growth factors and cytokines, they assume an activated, specialized myofibroblast phenotype that is the main source of CAFs [28,29,30]. These cells interact with other cell types such as inflammatory cells and endothelial cells, secreting ECM proteins, and matrix metalloproteases, thus ensuring the remodeling of the surrounding ecosystem. This favors the formation of new vessels, allowing metastatic spread into the microenvironment milieu [31,32,33,34,35,36,37]. Consistently with bioinformatics data, ECM deposition was not affected by Crenigacestat treatment, raising the speculation that the adjoining stroma serves as a physical barrier cooperating with cancer cells to prevent immune infiltration and promote immune escape, generating therapy-related resistant phenotypes. In this scenario, our results were also supported by IPA analysis, by Azan-Mallory staining, and by further analysis of α-SMA expression, both as RNA and protein levels and PDOs model. In conclusion, we hypothesize that the surrounding ecosystem plays a fundamental role in driving both drug responsiveness and tumor progression in iCCA. Moreover, a similar landscape of the tumor microenvironment is necessary for Crenigacestat effectiveness to modulate the tumor/host interaction, this being further supported by the demonstrated lack of drug effects on PDO-derived iCCA.

In a future perspective, the cross-talk between tumor and stromal cells will be the subject of future investigations in order to open out new directions in cancer therapy.

## 4. Materials and Methods

### 4.1. In Vivo Study

Animal experiments were carried out according to the protocol approved by the Ethics Committee (Prot. N. 254/C.E) at Biogem scarl Medicinal Investigation Research, MIR in Ariano Irpino, (Avellino, Italy), authorized to use animals in Biogem laboratories by the Italian Health Authority. The Care and Husbandry of animals are in accordance with European Directives n. 2010/63 and with the Italian Regulatory system (D.L. vo no. 26, 4 March 2014). CD-1 nude mice, females, 4–6 weeks old, were used for this study, subcutaneously injected into the right flank with 5 × 10^6^ HuCCT1 cells (CD90 negative). When the tumor masses reached the approximate volume of 60–70 mm^3^, 2–3 weeks after injection, mice were allocated to one of two experimental groups: one group treated orally (gavage) with the vehicle and the other treated orally (gavage) with Crenigacestat, administered 3 times/week for 4 consecutive weeks. All animals were weighed biweekly during the experimental period and sacrificed when the tumor masses were greater than 15% of body weight (BW) and/or body weight loss (BWL) was 10%. The BWL was determined as follows: % BWL max = 100 − (mean BWday x/mean BWday 1 × 100), where BWx is the mean BW on the day of maximal loss during the experiment and BW1 is the mean BW on the 1st day of the experimental period. In addition, the tumor volume formula (mm^3^) = [length (mm) × width (mm)^2^]/2 was applied, where width and length are the shortest and longest diameters. At the end of the experiment, the last dose of the drug or vehicle was administered 6 h before sacrifice, and the tumor masses were collected and preserved as follows: 1/2 frozen at −80 °C, 1/2 fixed in 4% formalin solution.

### 4.2. Establishment, Culture, and Treatment of Human iCCA-PDOs

The establishment and culture of PDOs from iCCA cancers were performed as previously described [38]. Briefly, fresh biopsy tissue from human iCCA was minced, conditioned in PBS/EDTA 5 mM for 15 min at room temperature, and digested in a fresh digestion solution containing 5 mL PBS/EDTA 1 mM in 2× TrypLe (Thermo Fisher Scientific, Waltham, MA, USA) for 1 h at 37 °C. Then, the dissociated cells in suspension were collected, and the remaining tissue was mechanically disrupted with a pipette to facilitate the release of other cells. All the cell suspension recovered was spun in Advanced DMEM/F12 (Thermo Fisher Scientific) at 1200 rpm, 5 min, 4 °C.

The pellet was resuspended in 120 µL of liquefied growth factor reduced (GFR) Matrigel (Corning, Glendale, AZ, USA), creating a single droplet at 4 °C and seeded in a 24-well tissue culture plate, warmed to 37 °C. Plates were incubated at 37 °C, 5% CO_2_ in cell culture incubator for 20 min to solidify Matrigel droplets and overlaid with complete human organoids media Advanced DMEM/F12 supplemented with 1× B27 additive (Thermo Fisher Scientific, Waltham, MA, USA), 1× N2 additive (Thermo Fisher Scientific, Waltham, MA, USA), 0.01% BSA (Roche, Basel, Switzerland), 2 mM L-Glutamine (Thermo Fisher Scientific, Waltham, MA, USA), 100 units/mL penicillin/streptomycin (Thermo Fisher Scientific, Waltham, MA, USA), and 13 cytokines: EGF (PeproTech, London, UK); Noggin (PeproTech, London, UK); R-Spondin 1 (PeproTech, London, UK); Gastrin (Sigma-Aldrich); FGF-10 (PeproTech, London, UK); FGF-basic (PeproTech, London, UK); Wnt-3A (R&D Systems, Minneapolis, USA); Prostaglandin E2 (Tocris Bioscience, Bristol, UK); Y-27632 (Sigma-Aldrich, Milano, Italy); Nicotinamide (Sigma-Aldrich, Milano, Italy); A83-01 (Tocris Bioscience, Bristol, UK); SB202190 (Sigma-Aldrich, Milano, Italy) HGF (PeproTech London, UK). Complete media were subsequently refreshed every two days.

### 4.3. Human iCCA-PDOs Treatment

Cultures were split when organoids became over-confluent. Matrigel was broken up mechanically with a pipette, and PBS-EDTA 1mM containing 1× TrypLe was then incubated for 20 min at 37 °C. Dissociated single cells were washed with HBSS (Thermo Fisher Scientific) and pelleted at 1200 rpm, 5 min, 4 °C. Briefly, 6 × 10^4^ cells were resuspended in GFR matrigel and seeded in standard 96-well cell culture plates with complete organoid media.

After 3 days post seeding, exhausted media were replaced by complete organoids media with DMSO or Crenigacestat at dilution series concentrations (0.15–10 µM). The drug-containing medium was changed every 2 days until the end of treatment. After 15 days of treatment, cell viability was assayed using CellTiter-Glo (Promega, Tokyo, Japan).

### 4.4. Protein Extraction and Western Blot Analysis

Total tissue proteins were extracted with tissue homogenizer using T-PER Tissue Protein Extraction Reagent containing 1:100 Halt Protease and Phosphatase Inhibitor Cocktail EDTA-free (Thermo Fisher Scientific). Proteins concentrations were quantified with Bradford Reagent (Bio-Rad, Hercules, CA, USA) using a BSA standard curve (0.0–40 μg/mL). The proteins were then mixed with Laemmli buffer and 10% β-mercaptoethanol (BME), denatured at 95 °C for 5 min, and loaded onto 4–20% polyacrylamide gradient gels and run on SDS-PAGE. After gel electrophoresis, the proteins were transferred onto nitrocellulose membranes (Trans-Blot Turbo Mini 0.2 μm Nitrocellulose Transfer Packs, Bio-Rad) using the Trans-Blot Turbo Transfer System (Bio-Rad). The blotted membranes were pre-incubated in 5% non-fat milk (Bio-Rad, Hercules, CA, USA) in Tris-buffered saline supplemented with 0.05% Tween-20 (TBS-T) for 1 h at room temperature and then incubated overnight at +4 °C with the following primary antibodies: human primary anti-Notch cleaved 1 (1:1000, Cell Signaling Technology, Pero, Italy); purified human anti-HES1 (1:1000, Cell Signaling Technology, Danvers, MA, USA); anti-CD31 (1:1000, Abcam, Cambridge, MA, USA), anti-DLL4 (1:1000, Abcam, Cambridge, MA, USA), anti-VEGFA (1:1000, Abcam, Cambridge, UK) and antiglyceraldehyde-3-phosphate dehydrogenase (GAPDH). After three washings with TBS-T, membranes were stained with the corresponding horseradish peroxidase-conjugated secondary antibodies diluted 1:5000 in TBS-T for 1 h at room temperature and subsequently washed once more with TBS-T. The chemiluminescence signal from proteins was revealed using Clarity Max Western ECL Substrate (Bio-Rad) and captured with the ChemiDoc MP instrument (Bio-Rad Laboratories) using Image Lab 5.2.1. The density mean values of the bands were calculated using Image J software. In the case of VEGFA, two outliers of the vehicle group were excluded from statistical data analysis.

### 4.5. RNA Extraction and cDNA Synthesis

Total RNA was extracted from the HuCCT1-xenograft model iCCA tissues using TRIzol^®^ reagent (Thermo Fisher Scientific) in combination with the TissueLyser homogenizer (Qiagen, Hilden, Germany), following the manufacturer’s instructions. To exclude DNA contamination, an additional column-based purification (RNeasy Mini Kit, QIAGEN) was used, including DNase digestion step. Total RNA concentrations and quality were determined with the Nano-Drop 2000/2000c (Thermo Fisher Scientific) and the Agilent 2100 Bioanalyzer (Agilent Technologies, Palo Alto, CA, USA). Specifically, RNA with 260/280 nm ratios ≥1.8 and RIN ≥8 was used for the transcriptomic analysis. One μg total RNA was reverse transcribed to cDNA using iScript cDNA Synthesis Supermix (Biorad, Hercules, CA, USA), according to the relative datasheet.

### 4.6. Gene Expression Analysis

Gene expression microarray was performed as already described [19], using the Affymetrix technology. Briefly, transcriptomic analysis was performed on RNA extracted from single fragments of each frozen tissue sample. The HTA2.0 Plus Reagents KIT was used to perform RNA retrotranscription, labeling, and hybridization. The operative instructions are those reported in the manual “GeneChipTM WT PLUS Reagent Kit Manual- Target Preparation for GeneChipTM Whole Transcript (WT) Expression Arrays” (Affymetrix). Hybridized arrays were automatically washed and stained by the GeneChipTM Fluidics Station 450 (Affymetrix) using the reagents indicated in the user manual. Then, chips were automatically acquired by the Affymetrix Complete GeneChip^®^ Instrument System Scanner, which generated both digital images (-DAT files) and raw intensity data (.CEL files). Quality control (QC) of the raw data was performed for evaluating samples quality, hybridization signal, and samples correlation. Further analyses on the samples were performed only if the raw data passed this initial QC. Internal controls were evaluated for data validation. The gene expression data are available under accession number GSE150024 at the Gene Expression Omnibus (http://www.ncbi.nlm.nih.gov/geo/ (accessed on 1 December 2021)).

### 4.7. Quantitative Real-Time PCR

Gene expression of MMP13, GAPDH, and ACTA2 was assessed using validated human primers: MMP13 Human PrimePCR™SYBR^®^ Green, Assay ID: qHsaCIP0026824 (Biorad); Hs_GAPDH_1_SG QuantiTect Primer Assay ID: QT00079247 (Qiagen) and primer sequences for ACTA2: forward, 5′-GGAATGGGACAAAAAGACAGCTA-3′; reverse, 5′-CGGGTACTTCAGGGTCAGGAT-3′. Comparative real-time PCR was performed in triplicate, including no-template controls. Real-time analysis was performed on a CFX96 System (Biorad, Hercules, CA, USA), and the 2^−ΔΔCt^ method was used to assess the relative expression.

### 4.8. Histological Staining and Scoring

For immunohistochemistry, frozen tissues sections were pre-fixed with 1:1 acetone–chloroform solution. α-SMA staining with antibody diluted 1:100 (Sigma-Aldrich) was performed with an automated autostainer (cat. K5007, Dako, Glostrup, Denmark). The sections were then observed using the Eclipse Ti2 microscope (Nikon Inc., Melville, NY, USA) and analyzed with Image J analysis software.

For Masson’s trichrome staining, all iCCA cryosections were fixed in 10% neutral buffered formalin for 30 min at room temperature, washed twice in distilled water for 5 min, and stained with Mallory trichrome acc. McFarlane kit (DIAPATH) following the manufacturer’s instructions. Briefly, Mallory’s trichrome staining protocol uses the three stains to reveal connective tissue, with a particular affinity for collagen, reticulum, cartilage, bones, and amyloid. After hydration, each section was covered with Acid Fuchsin 1% reagent for 1 min at room temperature. Sections were then quickly rinsed in distilled water and covered with Phosphomolybdic Acid 2% for 3 min at room temperature. After removing the reagent, the sections were covered with Mallory mixture for 30 s at room temperature, subsequently quickly rinsed in distilled water, dehydrated for a few seconds in ethyl alcohol 95°, absolute ethyl alcohol, and clarified with isoparaffin-based solvent (DIAPATH). Finally, the sections were mounted in balsam. The results of the histopathological samples were obtained the day after, and the images were acquired with the Eclipse Ti2 microscope (Nikon Inc., Melville, NY, USA) using an ×20 objective lens. The degree of fibrosis was classified as mild, moderate, or severe.

### 4.9. Statistical Analysis

For microarray analysis, the raw data were background-corrected and normalized with the robust multialignment algorithm (RMA) using the Affymetrix Expression Console. Differentially expressed genes (DEGs) between Crenigacestat-treated and vehicle-treated samples were identified with the Affymetrix Transcriptome Analysis Console 4.0 using the Limma eBayes method. DEGs were selected by applying a fold-change of ± 1.5 and *p*-value ≤ 0.05 (with or without FDR correction). Hierarchical clustering was generated using Alt Analyze 2.1.3 software [39]. Gene ontology analysis for biological processes was performed using GOrilla (Gene Ontology enRIchment anaLysis and visuaLizAtion tool) (http://cbl-gorilla.cs.technion.ac.il/ access on 15 December 2021) software [40].

In addition, Ingenuity Pathway Analysis (IPA) software (Qiagen, Germantown, MD, USA) was applied to identify the molecular networks and upstream transcriptional regulators modulated by DEGs, comparing Crenigacestat-treated and vehicle-treated samples.

Biological and technical triplicate results were analyzed with the appropriate statistical tests (unpaired t-test or ANOVA) in order to establish statistical significance and reproducibility. A *p*-value ≤ 0.05 was considered statistically significant. The values are presented as mean ± standard deviation (SD) or standard error of the mean (SEM). GraphPad Prism 5.0 software (La Jolla, CA, USA) was used for all statistical analyses.

## 5. Conclusions

**Figure 10:**
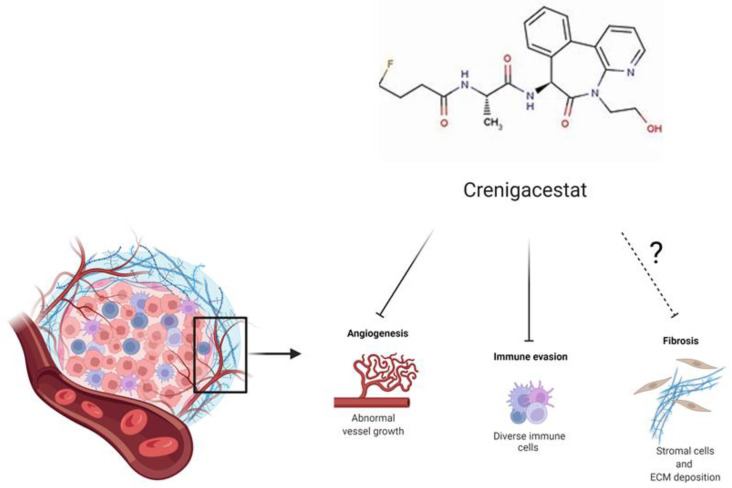
Schematic illustration of this study.

This study demonstrates that in a HuCCT1-xenograft mouse model, Crenigacestat specifically targets the expression of N1ICD and HES1. Beyond that, Crenigacestat is able to reduce the expression of CD31, DLL4, and VEGFA by blocking angiogenesis, whereas it does not affect fibrosis and does not down-regulate MMP13 likely because of its commitment to modulating the inflammatory response (Figure 10). These data highlight that the effect of Crenigacestat on the Notch signaling pathway depends on the cross-talk between tumor and stromal cells. HuCCT1-xenograft mice do not mimic the complexity of the microenvironmental conditions responsible for human iCCA tumor progression and treatment evasion. This underlines the importance of personalizing therapies in patients with cancer, and in particular those with iCCA, taking into account the desmoplastic reaction as a modulator of drug responsiveness and tumor progression.

## Figures and Tables

**Table 1:** The 24 top-ranked networks modulated by Crenigacestat.

ID	Top Diseases and Functions	Score	Focus Molecules
1	Cancer, gastrointestinal disease, organism injury and abnormalities	55	31
2	Cancer, cell morphology, tissue development	38	24
3	Cell morphology, embryonic development, hair and skin development and function	33	22
4	Metabolic disease, protein degradation, protein synthesis	33	22
5	Carbohydrate metabolism, cardiovascular disease, post-translational modification	31	21
6	Cell death and survival, lipid metabolism, nervous system development and function	31	21
7	Cellular development, cellular growth and proliferation, cellular movement	29	20
8	Dermatological diseases and conditions, immunological disease, organismal injury and abnormalities	25	18
9	Developmental disorder, organism injury and abnormalities, renal and urological disease	23	17
10	Cancer, cell cycle, gene expression	23	17
11	Embryonic development, hematological system development and function, lymphoid tissue structure and development	21	16
12	Cancer, dermatological diseases and conditions, organism injury and abnormalities	21	16
13	Cellular assembly and organization, lipid metabolism, small molecule biochemistry	21	16
14	Auditory disease, hereditary disorder, neurological disease	21	16
15	Dermatological diseases and conditions, immunological disease, inflammatory disease	19	15
16	Cell morphology, embryonic development, nervous system development and function	19	15
17	Cancer, cellular assembly and organization, connective tissue disorders	18	14
18	Cell morphology, organ morphology, organism injury and abnormalities	16	13
19	Drug metabolism, increased levels of AST, lipid metabolism	14	12
20	Cardiovascular disease, cellular compromise, organism injury and abnormalities	14	12
21	Embryonic development, organism development, tissue morphology	13	11
22	Cell cycle, cellular movement, infectious diseases	13	11
23	Connective tissue development and function, connective tissue disorders, organism injury and abnormalities	8	8
24	Cellular movement, immune cell trafficking, inflammatory response	7	7

## Supplementary Materials

The following supporting information can be downloaded at: https://www.mdpi.com/1422-0067/23/8/4187/s1.

## Abbreviations

CAFs	Cancer-associated fibroblasts
DLL4	Delta-like canonical Notch ligand 4
ECM	Extracellular matrix
GO	Gene ontology
GSI	Gamma secretase inhibitor
HCC	Hepatocellular carcinoma
HES1	Hairy/enhancer of split 1
iCCA	Intrahepatic cholangiocarcinoma
IPA	Ingenuity pathway analysis
NICD	Notch intracellular domain
PDX	Patient-derived xenograft
VEGFA	Vascular endothelial growth factor A
